# Case Report: Immune-driven clonal selection underlying lineage switch from B-Precursor acute lymphoblastic leukemia to acute myeloid leukemia following inotuzumab ozogamicin

**DOI:** 10.3389/fimmu.2026.1828617

**Published:** 2026-07-29

**Authors:** Kaori Kondo, Daichi Sadato, Yuho Najima, Toshikazu Itabashi, Takahiro Ueda, Chizuko Hirama, Masashi Shimabukuro, Atushi Jinguji, Naoki Shingai, Takashi Toya, Hiroaki Shimizu, Tomomi Toubai, Hironori Harada, Yuka Harada, Noriko Doki

**Affiliations:** 1 Hematology Division, Tokyo Metropolitan Cancer and Infectious Diseases Center, Komagome Hospital, Tokyo, Japan; 2 Clinical Research and Trials Center, Tokyo Metropolitan Cancer and Infectious Diseases Center, Komagome Hospital, Tokyo, Japan; 3 Department of Pediatrics, Nippon Medical School Hospital, Tokyo, Japan; 4 Laboratory of Oncology, School of Life Sciences, Tokyo University of Pharmacy and Life Sciences, Tokyo, Japan

**Keywords:** acute Myeloid Leukemia, B-precursor acute lymphoblastic leukemia, clonal analysis, inotuzumab ozogamicin, lineage switch

## Abstract

Lineage switch (LS), defined as a change in leukemic lineage during the disease course, is a rare but clinically significant event in acute leukemia and is typically associated with poor prognosis. Although LS has been increasingly reported following targeted immunotherapies, the clonal mechanisms underlying this phenomenon remain incompletely understood, particularly in cases without KMT2A rearrangement. We report a case of LS from B-precursor acute lymphoblastic leukemia (BCP-ALL) to acute myeloid leukemia (AML) following treatment with the CD22-targeted antibody–drug conjugate inotuzumab ozogamicin. To elucidate the clonal architecture underlying LS, targeted next-generation sequencing was performed on bone marrow samples obtained at multiple time points throughout the disease course. Genomic analysis demonstrated that the lymphoid and myeloid disease phases shared ancestral genetic alterations but displayed distinct mutational profiles. At the time of LS, TP53 and SMC1A mutations newly emerged, whereas only a subset of mutations detected at ALL relapse was retained. These findings suggest that the AML phase most likely resulted from the selective expansion of a genetically distinct subclone derived from a common progenitor, rather than the direct transdifferentiation of the dominant ALL clone, consistent with immunotherapy-driven clonal selection. Longitudinal genomic profiling revealed stepwise clonal evolution during disease progression, supporting a model of immunotherapy-driven clonal selection leading to LS. This case provides molecular evidence suggesting that immune-targeted therapy can promote expansion of minor pre-existing subclones with alternative lineage potential within a common progenitor even in non-KMT2A-rearranged leukemia. Our findings highlight the importance of comprehensive genomic monitoring during immunotherapy to identify therapy-resistant subclones and better understand mechanisms of lineage plasticity in acute leukemia.

## Introduction

Lineage switch (LS) is a rare phenomenon in which the phenotype of a hematological malignancy changes to another lineage during the clinical course while maintaining cytogenetic and/or molecular features of the original disease, typically resulting in poor prognosis (1, 2). Several mechanisms have been proposed, including direct transdifferentiation, dedifferentiation followed by redifferentiation into another lineage, and phenotypically distinct clonal replacement among cells derived from a common progenitor but committed to different lineages; however, the precise mechanism remains unclear (3). Recently, selective pressure from targeted immunotherapies, including blinatumomab, chimeric antigen receptor T-cells, and antibody-drug conjugates such as inotuzumab ozogamicin, has been implicated in LS (4, 5). Although LS has been well characterized in leukemia with *KMT2A* rearrangement (*KMT2A*r), its clinical features in non-*KMT2A*r cases remain poorly defined (2).

Here, we report a case of LS from B-cell precursor acute lymphoblastic leukemia (BCP-ALL) to acute myeloid leukemia (AML) in a patient without *KMT2A*r, following inotuzumab ozogamicin (InO) therapy. To elucidate the clonal architecture and evolutionary dynamics, we performed targeted sequencing at multiple time points throughout the treatment course. Our analysis revealed the emergence of genetically and phenotypically distinct clones originating from a common ancestral clone, supporting LS through clonal replacement.

## Case description

**Figure 1:**
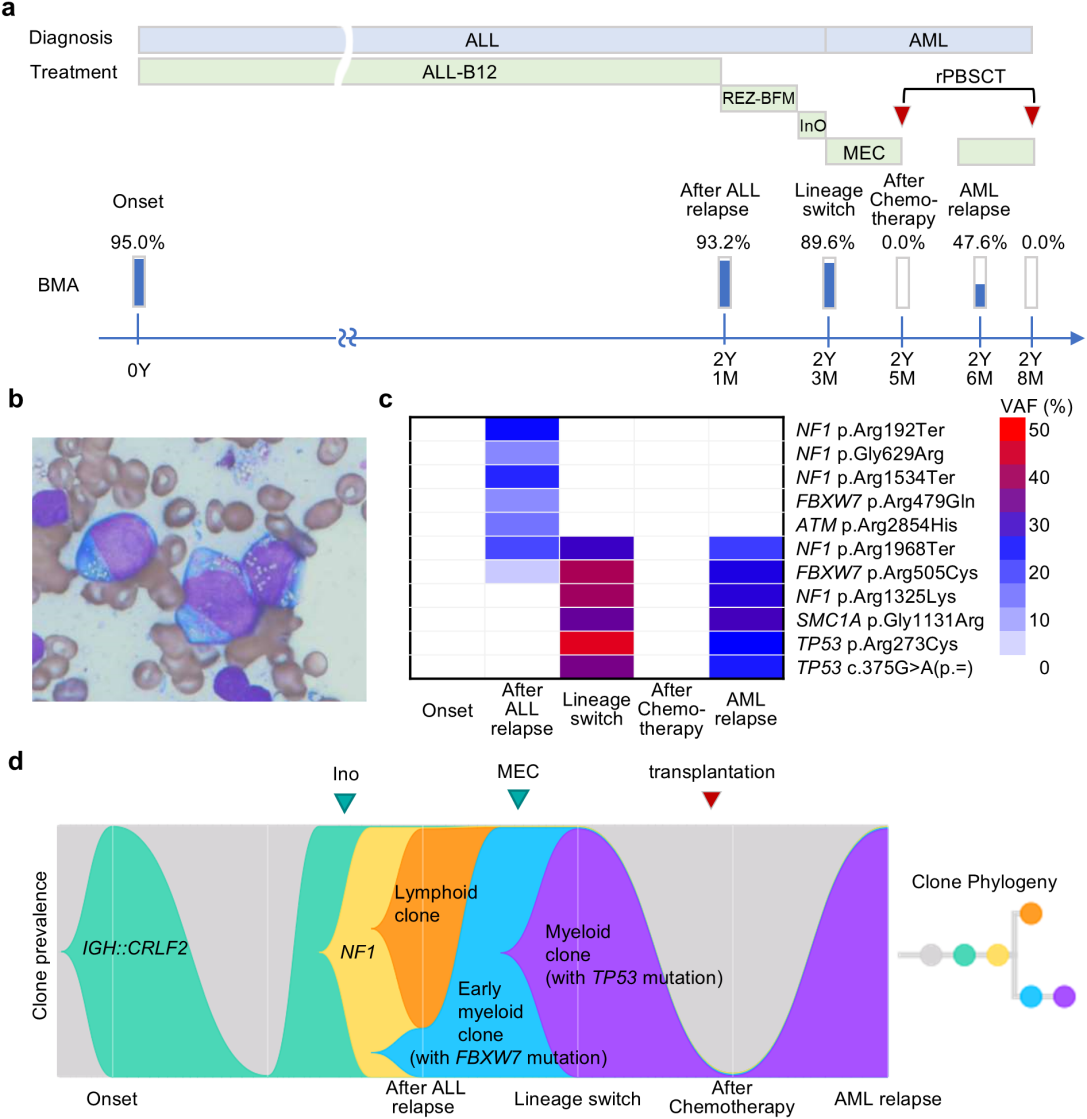
**(a)** Clinical disease course. ALL, acute lymphoblastic leukemia; AML, acute myeloid leukemia; BMA, bone marrow aspiration; InO, inotuzumab ozogamicin; M, month; rPBSCT, related peripheral blood stem cell transplantation; Y, year. One course of MEC chemotherapy consisted of mitoxantrone (6 mg/m^2^ on days 1–6), etoposide (80 mg/m^2^ on days 1–6), and cytarabine (1 g/m^2^ on days 1–6) administered intravenously. **(b)** Bone marrow aspirate smear at the lineage switch phase (Giemsa stain). **(c)** Mutational profiles based on targeted sequencing. Color intensity corresponds to the variant allele frequency of each mutation. **(d)** Fish plot illustrating inferred clonal evolution of leukemic cells based on targeted sequencing data.

The clinical course of the patient is summarized in the timeline shown in **Figure 1a**. A 19-year-old male was diagnosed with BCP-ALL. Bone marrow (BM) examination revealed 95% leukemic blasts. Flow cytometric (FCM) analysis showed a B-lineage immunophenotype (CD19+, CD10+, cyCD22+), with negativity for cyCD3, cyMPO, CD14, and CD64. FCM also demonstrated high CRLF2 expression on leukemic blasts. Myeloperoxidase (MPO) immunohistochemical staining was negative. Cytogenetic analysis revealed a karyotype of 47, XY, +X [15/20] and 46, XY [5/20]. Real-time PCR screening did not detect *KMT2A*r (*KMT2A::AF4*, *KMT2A::AF6*, *KMT2A::AF9*, or *KMT2A::ENL*). The patient achieved complete remission (CR) following induction therapy based on the JPLSG ALL-B12 protocol. During maintenance therapy, the patient experienced relapse with recurrent BM involvement and a similar B-lineage immunophenotype. Karyotype analysis revealed 47, XY, +X, del(1)(p)?, t(9;11)(p13;q23.3) [19/20] and 46, XY [1/20]. Molecular testing confirmed the absence of *KMT2A*r. FCM at relapse again demonstrated high CRLF2 expression. In addition, FISH revealed split signals of both *IGH* and *CRLF2*, suggesting that an *IGH::CRLF2* rearrangement may have been present from diagnosis. Salvage therapy with the ALL-REZ-BFM 2002 protocol failed, and inotuzumab ozogamicin (InO) was administered at doses of 0.8 mg/m^2^ on day 1 and 0.5 mg/m^2^ on days 9 and 15. BM examination after 22 days revealed 90% blasts with fine nuclear chromatin and cytoplasmic vacuolation (**Figure 1b**). FCM analysis demonstrated a complete immunophenotypic shift characterized by loss of B-lineage markers and acquisition of myeloid markers (CD11c and CD64), while CD3 remained negative (**Table 1**). Immunohistochemical staining revealed MPO positivity. Cytogenetic analysis revealed 47, XY, +X, der(1), del(1)(P?)?ins(1;?)(q23;?), t(9;11)(p13;q23.3) [19/20] and 46, XY [1/20]. Abnormalities such as +X, del(1)(p?) and t(9;11)(p13;q23.3) were consistent with those observed at the prior ALL relapse phase, supporting a diagnosis of LS rather than lineage drift from B-lymphoid to myeloid leukemia, as defined by a recent large cohort study (2). Following cytoreduction with hydroxycarbamide, the patient received MEC chemotherapy consisting of etoposide (80 mg/m^2^/d), cytarabine (1000 mg/m^2^/d), and mitoxantrone (6 mg/m^2^/d) for 6 d and achieved hematological CR. After bridging therapy with venetoclax (50 mg/d combined with posaconazole) and azacitidine (75 mg/m^2^/d for 5 d), the patient underwent HLA haploidentical peripheral blood stem cell transplantation (PBSCT) from his mother. Conditioning consisted of fludarabine (30 mg/m^2^/d for 6 d), cytarabine (2000 mg/m^2^/d for 4 d), melphalan (70 mg/m^2^/d for 2 d), and 4 Gy total body irradiation. Graft-versus-host disease prophylaxis included antithymocyte globulin, tacrolimus, and methylprednisolone (1 mg/kg/d). BM aspiration on day 26 post-PBSCT revealed 47.6% blasts. FCM analysis showed a myeloid immunophenotype, with loss of B-lineage markers (CD19, CD10, and CD22) and expression of CD11c, while CD14 remained negative. MPO staining was positive. Cytogenetic analysis showed 47, XY, +X, der(1), del(1)(p)??ins(1);?(q23);?, add(9)(p13)add(11)(q24) [10/12] and 46, XX [2/12]. Accordingly, the patient was diagnosed with relapsed AML post-PBSCT. The patient received MEC chemotherapy again and achieved a second hematological CR, although a small number of atypical blast cells persisted in the BM. Following high-dose cytarabine (2000 mg/m^2^/d for 4 d), a second conditioning regimen consisting of fludarabine (30 mg/m^2^ for 3 d) and 12 Gy total body irradiation was administered, followed by a second HLA-haploidentical PBSCT from his older sister. Post-transplant cyclophosphamide (40 mg/kg on days three and four), tacrolimus, and mycophenolate mofetil were used as graft-versus-host disease prophylaxis. Although no circulating blast cells were detected following the second transplantation, the patient died on day 41 from severe sinusoidal obstructive syndrome complicated by transfusion-refractory thrombocytopenia and fatal nasopharyngeal hemorrhage. The clinical course is summarized in **Figure 1a**.

**Table 1:** Changes over time since onset in cell surface antigens, chromosomes, genetic variation, and gene expression.

	Onset	ALL relapse	Lineage switch to AML	AML relapse
FCM					
B lineage	CD19+, CD10+, CD22+, CD24+, cyCD79a+; CD20-	CD19+, CD10+, CD22+, CD24+, cyCD79a+; CD20-	CD19-, CD10-, CD22-, CD24-, cyCD79a-, CD20-	CD19-, CD10-, CD22-
T lineage	cyCD3-, CD3-	cyCD3-, CD3-	cyCD3-, CD3-	cyCD3-, CD3-
Myeloid	CD33+; cyMPO-, CD13-, CD14-, CD15-, CD64-, CD65-	cyMPO-, CD13-, CD14-, CD15-, CD33-, CD64-, CD65-	CD15+, CD33+, CD64+, CD117+; CD13-, CD14-	CD15+, CD33+ CD13(dim)+, CD117(dim)+
CRLF2	Positive	Positive	N.A.	N.A.
MPO (cytochemistry)	Negative	Negative	Positive	Positive
G-banding	47,XXY	47,XXY, del(1)(p?),t(9;11)(p13;q23.3)	47, XXY, der(1)del(1)(p?)?ins(1;?) (q23;?), t(9;11)(p13;q23.3)	47, XXY, der(1)del(1)(p?)? ins(1;?)(q23;?), add(9)(p13) add(11)(q24)
Gene rearrangements (FISH / qPCR)	*KMT2A* rearrangement: Negative	*KMT2A* rearrangement: Negative *CRLF2* split: Positive IGH split: Positive	*KMT2A* rearrangement: Negative	*KMT2A* rearrangement: Negative
DNA targeted sequencing	No pathogenic variants detected	*NF1*, *KRAS*, *FBXW7*, *ATM*	*NF1*, *FBXW7*, *SMC1A*, *TP53*	*NF1*, *FBXW7*, *SMC1A*, *TP53*
Copy number alterations	*IKZF1* deletion, *chr X* amplification	*IKZF1/CDKN2A/CDKN2B* deletion, chr X amplification	*IKZF1/CDKN2A/CDKN2B /VPREB1* deletion, chr X amplification	*IKZF1/CDKN2A/CDKN2B /VPREB1/CEBPA* deletion, chr X amplification
RNA targeted sequencing	High expression: *BAALC*	High expression: *BAALC*, *WT1*	High expression: *BAALC*, *WT1*, *MECOM*	High expression: *WT1*, *MECOM*

ALL, acute lymphoblastic leukemia; AML, acute myeloid leukemia; CD, cluster of differentiation; cyCD3, cytoplasmic cluster of differentiation 3; cyCD79a, cytoplastic cluster of differentiation 79a; cyMPO, cytoplasmic myeloperoxidase; dim, low-intensity expression by flow cytometry; FCM, flow cytometry; FISH, fluorescence in situ hybridization; MPO, myeloperoxidase; N.A., not assessed; qPCR, quantitative polymerase chain reaction.

Targeted sequencing was performed to delineate the clonal architecture and genetic transitions associated with LS. BM specimens collected at five time points: at diagnosis, ALL relapse, myeloid LS, after the first MEC treatment, and AML relapse, were analyzed. Sequencing and analysis methods have been described previously (6, 7). All detected genetic alterations are listed in **Supplementary Table 1** and summarized in **Table 1** and **Figure 1c**, and the inferred clonal evolution model is shown in **Figure 1d**. At ALL relapse, mutations in *NF1*, *KRAS*, *FBXW7*, and *ATM* were identified. Concurrent with these genetic findings, the B-lineage immunophenotype and high CRLF2 expression remained preserved, consistent with the initial presentation. At the time of myeloid LS, most mutations detected at ALL relapse were absent; however, new *TP53* and *SMC1A* mutations emerged, with retention of *NF1* p.Arg1968Ter and *FBXW7* p.Arg505Cys. The increased variant allele frequencies (VAFs) of *NF1* p.Arg1968Ter and *FBXW7* p.Arg505Cys suggested selective expansion of a specific subclone. At AML relapse following the first transplantation, the mutation profile remained largely unchanged compared with that observed in myeloid LS, supporting clonal stability after lineage transition. Copy number analysis showed persistent *IKZF1* deletion and amplification of genes on the X chromosome across all analyzed time points, indicating a shared clonal origin. In contrast, *CDKN2A/B* deletion was acquired at ALL relapse, *VPREB1* deletion emerged at myeloid LS and was retained thereafter, and *CEBPA* loss was detected only at AML relapse, indicating a stepwise acquisition of genetic alterations during disease progression.

Collectively, the presence of shared mutations alongside distinct genetic alterations in ALL- and AML-phenotype clones indicated that these populations were not identical, but rather represented divergent subclones derived from a common progenitor. Subsequent LS was likely driven by therapeutic selective pressure, particularly immunotherapy.

This case offers critical insights into LS pathophysiology via longitudinal targeted sequencing. Few reports provide comprehensive clonal analysis throughout LS (**Supplementary Table 2**). In *KMT2A*r cases, epigenetic plasticity typically facilitates phenotypic lineage switching (2, 8). Similar mechanisms are proposed for non-*KMT2A*r cases where conserved genetic alterations indicate a persistent dominant founder clone. In contrast, our non-*KMT2A*r case exhibited marked genetic divergence between the two disease phases. Since only specific subclones harboring *NF1* and *FBXW7* mutations were shared, our findings support a model where minor subclones branched from a common progenitor, expanded under therapeutic selective pressure, and replaced the dominant clones during LS. This phenotypically distinct clonal replacement and selection model aligns with recent large-scale analyses of post-immunotherapy LS (2). Our data clarify the timing and pattern of clonal emergence and transition, highlighting the importance of comprehensive clonal assessment both before and after therapeutic interventions.

LS is associated with a poor prognosis (1, 2), and no standard treatment strategy has been established. Recent studies have highlighted the role of immunotherapy-driven selective pressure in unmasking therapy-resistant subclones (2), thus, single-agent immunotherapy warrants caution. In our case, MEC-based cytotoxic chemotherapy achieved complete hematological remission after myeloid LS, indicating that the lineage-switched disease retained chemosensitivity. These findings suggest that early identification of subclonal heterogeneity by next-generation sequencing (NGS) may guide treatment by detecting therapy-resistant subclones before or at LS. Moreover, future studies utilizing single-cell sequencing or clonal tracking across different hematopoietic compartments (9) will be essential to clarify whether the alternative lineage program was already stably encoded in the ancestral progenitor prior to therapy. In this evolving therapeutic landscape, treatment strategies may need to diverge according to the molecular context. Acute leukemia with *KMT2A*r may benefit from targeted therapy with menin inhibitors (10), whereas non–*KMT2A*r cases lack established lineage-specific targeted therapies. Therefore, our case supports the consideration of combination or sequential approaches, including the integration of cytotoxic chemotherapy with immunotherapy such as blinatumomab or InO, rather than reliance on immunotherapy alone (2). Consistent with this notion, a previous report (2) demonstrated that InO was the most proximal ALL therapy in 5.7% (4/70) of LS cases, supporting its role as a rare but established trigger of LS.

Notably, the lineage-switched AML in this case harbored biallelic *TP53* alterations, a molecular feature consistently associated with profound treatment resistance and dismal outcomes, even following allogeneic transplantation, as demonstrated in previous studies (11–13). This observation highlights the clinical value of longitudinal NGS, as the emergence or expansion of *TP53*-mutated clones at relapse may identify patients at exceptionally high risk of treatment failure.

Our study had several limitations. First, as a single-case observation, the relative contributions of clonal replacement and immunotherapy-driven selective pressure to LS cannot be definitively determined, although the longitudinal genomic data support immunotherapy-associated clonal selection (2). Second, the absence of systematic clonal tracking such as *CRLF2* may have reduced the resolution of clonal continuity (8). Nevertheless, the identification of shared ancestral mutations across disease phases supports the concept that phenotypic divergence arose from underlying genotypic branching. Finally, the treatment responses observed here may not be generalizable.

The patient’s family provided written informed consent for publication and expressed hope that this report may contribute to a better understanding of rare disease evolution in acute leukemia.

In conclusion, our findings suggest that LS can arise through molecular clonal replacement from a common ancestral origin under immunotherapy-driven selective pressure. Furthermore, our data highlight the importance of delineating clonal architecture, including multilineage and genotype-defined disease states, to guide optimal treatment selection. These findings underscore the importance of longitudinal genomic monitoring during immunotherapy to detect emerging therapy-resistant subclones.

## Data Availability

The datasets presented in this article are not publicly available because they contain information that could compromise the privacy of the participant. The datasets are available from the corresponding author on reasonable request.
